# Fiscal agricultural expenditures’ impact on sustainable agricultural economic development: Dynamic marginal effects and impact mechanism

**DOI:** 10.1371/journal.pone.0299070

**Published:** 2024-02-29

**Authors:** Shengfang Zhang, Xiaodong Zhang

**Affiliations:** 1 School of Public Finance and Taxation, Dongbei University of Finance and Economics, Dalian, Liaoning, China; 2 Department of Public Works, Yantai Huangbohai New District Construction and Transport Bureau, Yantai, Shandong, China; National Technical University of Athens: Ethniko Metsobio Polytechneio, GREECE

## Abstract

Sustainable agricultural economic development is the core task for achieving the objective of rural revitalization strategy in China, which cannot be separated from the support and guidance of fiscal policy, and agricultural industry integration is a key path for the fiscal promotion of sustainable agricultural economic development. This paper systematically examines the interaction mechanism between fiscal agricultural expenditures and sustainable agricultural economic development by using 31 provincial panel data in China from 2008 to 2020 and adopting a two-way fixed effect model, a panel quantile model, and a mediating effect model, respectively. The results show that the impact of fiscal agricultural expenditures on sustainable agricultural economic development is significantly positive, and appears a dynamic increasing trend with the agricultural development stage upgrading. Moreover, heterogeneity analysis shows that the effect of fiscal agricultural expenditures is more obvious for the samples in the central region and with a high share of primary industry. Further, a mediating effect test finds that agricultural industry integration plays a mediating mechanism between fiscal agricultural expenditures and sustainable agricultural economic development. Therefore, this paper proposes constructing a long-term investment mechanism for fiscal agricultural expenditures, formulating differentiated fiscal support policies for agriculture, and prioritizing support for agricultural industry integration, which provides theoretical support and policy inspiration for promoting sustainable agricultural economic development.

## 1. Introduction

Agriculture is the foundation of the national economy and is the core and source of China’s rural revitalization. In 2023, the No.1 document of the Central Committee of the Communist Party once again clarifies the goal orientation based on the strategic height of comprehensively promoting rural revitalization to build a strong agricultural country with strong supply guarantee, strong scientific and technological equipment, strong management system, strong industrial resilience, and strong competitive ability. The policy orientation of national development confirms the importance of the status of agriculture, and accelerating sustainable agricultural economic development is a strategic choice to meet the needs of the situation. However, for a long time, China’s agricultural development has been characterized by widespread and persistent problems such as low productivity and low level of industrial development [1], which has seriously hindered the pace of rapid agricultural development. At present, it is urgent to find effective development momentum for sustainable agricultural economy, and it is necessary to seek a breakthrough from the critical path of re-examining the development of the agricultural economy. Fiscal agricultural expenditures and agricultural industry integration are two main forces to promote sustainable agricultural economic development, thus fully releasing the potential efficacy of the two is precisely the remedy to break the shackles of agricultural development in China.

Fiscal agricultural expenditure is an important embodiment of fiscal function to optimize resource allocation in the field of agriculture [2]. The fundamental status of agriculture, its positive external attributes, and its weak characteristics determine the necessity and importance of fiscal support [3]. As an important financial guarantee for deepening agricultural development, the investing scope of fiscal agricultural expenditures covers the key elements of agricultural pre-production, production, and post-production, including agricultural infrastructure construction, agricultural technology research and promotion, agricultural structural adjustment, inclusive financial development, and other aspects. With China’s rural revitalization strategy, fiscal agricultural expenditures have also been given new requirements to drive agricultural economic growth more accurately, comprehensively, and efficiently: In the direction of support, more attention has been paid to the investment in the construction of a modern agricultural economic system consisting of three major systems of modern agricultural industry, production and operation. In the mode of support, more emphasis has been placed on the diversified supply of fiscal policies, efficient utilization of financial funds, and the full play of financial leverage.

Agricultural industry integration is the core path to enhancing the overall competitiveness of the agricultural industry. It is based on agriculture, breaks the industrial boundaries through multi-dimensional channels such as internal integration of agriculture, the extension of agricultural industry chain, and expansion of agricultural functions, realizes the integration and penetration of agriculture and secondary and tertiary industries, and has an important driving effect on sustainable agricultural economy [4,5]. Therefore, agricultural industry integration, as a core element of building a modern agricultural economic system [6], is inevitably one of the key paths of fiscal support for agriculture to promote agricultural production, value-added, and efficiency, and should become a key investment target of fiscal agricultural expenditures. Agricultural industry integration gets strong financing support in fiscal agricultural policy, while fiscal agricultural expenditures provide strong kinetic energy for agricultural economic development by guaranteeing financial investment in agricultural industry integration and realizing the precise and efficient utilization of limited fiscal funds.

Nowadays, from the macro policy level, China has provided favorable fiscal policy support for the implementation of the rural revitalization strategy in terms of top-level design and institutional arrangements, and theoretically, fiscal agricultural policy has a key incentive effect on sustainable agricultural economic development; however, from the micro practice level, the use of fiscal agricultural expenditures is not sufficiently effective in matching rural revitalization strategy, and problems such as fragmentation of fiscal funds management mode and insufficient guidance of social capital are particularly prominent [7,8], which impede the realization of fiscal agricultural policies. Furthermore, confined to the fact that China’s agricultural foundation is still weak, the radiation-driven ability and guiding effect of agricultural industry integration main bodies are generally weak, and the problems of financing difficulty, expensive financing, and slow financing that they face are particularly prominent [9], leading to the suppression of the potential utility of agricultural industry integration, which seriously hinders China’s agricultural development.

As a result, the relationship between fiscal agricultural expenditures and sustainable agricultural economic development is still in some doubt: Have fiscal agricultural expenditures effectively promoted sustainable agricultural economic development? Does the important path of agricultural industry integration play an intermediary role? Hence, the relationship among fiscal agricultural expenditures, agricultural industry integration, and sustainable agricultural economic development needs to be further sorted out. Given this, this paper takes the data of 31 provinces in China from 2008 to 2020 as an example. Firstly, adopts the two-way fixed effect model to explore the average impact of fiscal agricultural expenditures on sustainable agricultural economic development. Secondly, constructs the panel quantile model to explore the marginal effect of fiscal agricultural expenditures on sustainable agricultural economic development and its evolution trajectory. Finally, applies the mediating effect model to test the impact mechanism and analyze the role of agricultural industry integration between fiscal agricultural expenditures and sustainable agricultural economic development.

The main contributions of this study are as follows. First, in terms of research methodology, this paper uses the panel quantile method to explore the dynamic marginal effect of fiscal agricultural expenditures at different agricultural development stages, which reveals the non-linear relationship between fiscal agricultural expenditures and sustainable agricultural economic development. Second, in terms of research perspective, this paper proposes and tests agricultural industry integration as a mediating variable, which broadens the study on the transmission mechanism of fiscal agricultural expenditures affecting sustainable agricultural economy. Third, the research conclusion interprets the mediating mechanism of agricultural industry integration, provides new ideas and perspectives for improving fiscal agricultural policies, and offers empirical evidence to take full advantage of fiscal agricultural expenditures and promote sustainable development of the agricultural economy.

This paper is organized as follows. Section 2 introduces the literature review and theoretical hypotheses. Section 3 presents the research design. Section 4 discusses the regression results. Section 5 further analyzes the impact path of fiscal agricultural expenditures on sustainable agricultural economic development. Section 6 provides the conclusions and policy implications.

## 2. Literature review and theoretical framework

### 2.1 Literature review

Fiscal agricultural expenditure is regarded as an important source of funding for agricultural development. The existing literatures based on macro data from countries around the world show there is a close relationship between fiscal agricultural expenditures and sustainable agricultural economic development.

Several studies have confirmed that fiscal agricultural expenditures have a positive role in promoting sustainable growth in agricultural economy, and the multiple effects of fiscal agricultural expenditures are discussed from the view of the multi-dimensional connotation of sustainable agricultural economic development. First, for the impact on sustainable agricultural production, Uthes et al. [10] evaluate the cost-benefit of agricultural policy measures guided by the sustainable development strategy of the EU, finding that agricultural public expenditures have a significant effect on agricultural economic growth in general. Through a multi-country empirical study in Southern Africa, Matchaya [11] finds that fiscal agricultural expenditures have a more obvious effect on the promotion of animal husbandry, fishery, and other easily neglected fields, and it is necessary to attach importance to the investment allocation of various industries within agriculture. Kamenya et al. [12] assess the impact of public agricultural expenditures on food security using data from Western African countries as an example, concluding that fiscal funds are effective in improving food availability.

Second, for the impact on sustainable agricultural society, Bollman and Ferguson [13] find that fiscal subsidy cancellation has negative spillover effects on the local non-agricultural economy, leading to significant reductions in farm value-added, farm asset value, and local non-farm employment. With statistical models, Diallo and Wouterse [14] and Sánchez et al. [15] simulate the potential impacts of fiscal agricultural investment and conclude that public investment led by agricultural productive infrastructure will significantly promote agricultural growth, thereby reducing dependence on food imports, raising income from food production and reducing poverty, which is conducive to economic recovery after the COVID-19 pandemic.

Third, for the impact on sustainable agricultural environment, Zafeiriou et al. [16] believe that effective fiscal policy tools will help agricultural enterprises mitigate greenhouse gas effects by limiting negative externalities, thus ensuring ecological efficiency and promoting the sustainable development of environmentally friendly agricultural industries. Kour et al. [17] point out that funding for crop nutrients such as fertilizers is an important part of sustainable agricultural environment. Koutroumanidis et al. [18] show that fiscal investment policies protect, improve the stock structure, and increase forest through a price transmission mechanism, thus promoting agricultural environmental sustainability. Krishnan et al. [19] propose that government interventions such as fiscal and tax incentives for sustainable processes and consumption taxes on non-renewable resources can help improve the efficiency of agricultural operations and resource recovery to achieve agricultural environmental sustainability. According to Melchior and Newig [20], agricultural systems co-evolve with many dimensions such as environment and institutions, and public funding for sustainable agriculture and reform of counterproductive incentive systems are specific policy levers to promote the transformation of agriculture to sustainable development.

Conversely, some studies have indicated that fiscal agricultural incentives may have negative effects. Through a study of the agricultural policy subsidies effects in the EU, Latruffe et al. [21] find that the effect of agricultural subsidies on technical efficiency can be either positive, no effect, or even negative, which depends on the specific type of subsidy in a particular country. Garrone et al. [22] argue that although the combined effect of agricultural subsidies is to promote agricultural labor productivity growth, this combined effect masks important heterogeneity in various subsidies’ effects, some of which produce outcomes that slow agricultural productivity. Li and Xie [23] prove that Chinese local governments, in the interest game of soft budget constraints, have the phenomenon of blindly expanding fiscal investment that does not meet the needs of the agricultural economy and that this kind of crude agricultural support will lead to the long-term fiscal inefficiency, and will not be able to significantly improve agricultural economic efficiency. Although Zhang and Ouyang [24] affirm the positive impact of China’s fiscal policy on agricultural development, i.e., stimulating farmers’ enthusiasm for planting and thus increasing the total value of agricultural output, they also point out the bias of the policy effect, i.e., leading to over-reliance on the expansion of the cultivated area and the excessive application of pesticides, which further result in the low quality of agricultural products, the imbalance of the supply structure, and the destruction of the agricultural environment, which is detrimental to the agricultural development quality.

From previous literatures, it can be seen that fiscal agricultural expenditures have the potential positive effect of promoting sustainable agricultural economic development in theory, but its real impact effect is uncertain. In general, the existing academic achievements have enriched the research related to the effect of fiscal agricultural expenditures on sustainable agricultural economy and put forward more valuable academic views, but there are still issues worthy of further in-depth discussion. Firstly, the existing literatures mostly focus on the linear relationship between fiscal agricultural expenditures and agricultural economy, examining the average impact of fiscal agricultural expenditures, while it rarely considers the potential non-linear correlation between the two, and has not explored the heterogeneity in the development of agricultural economy. Secondly, few scholars have explored the influencing mechanism of fiscal agricultural expenditures on sustainable agricultural economic development, and even fewer documents have included fiscal agricultural expenditures, agricultural industry integration, and sustainable agricultural economic development into the same research framework, to demonstrate the relationship among the three, and to test whether agricultural industry integration has played a significant intermediary role.

Therefore, to enrich the existing research, this paper tries to fill this gap through empirical research on Chinese provincial data and proposes a path to strengthen the sustainable development of the agricultural economy in the fiscal policy, which has certain theoretical innovation and strong practical significance.

### 2.2 Theoretical framework

#### 2.2.1 Relationship between fiscal agricultural expenditures and sustainable agricultural economic development

**Fig 1:**
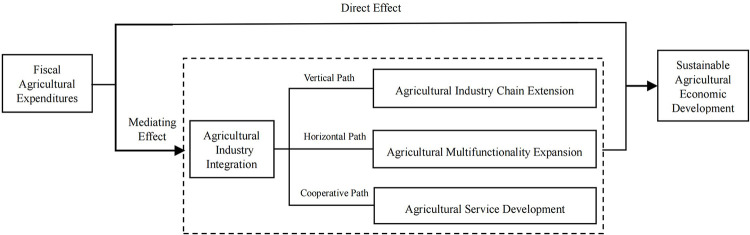
Mechanism of fiscal agricultural expenditures affecting sustainable agricultural economic development.

The weakness and externality characteristics of agriculture and its basic position in the national economy jointly determine the necessity and importance of fiscal support for agriculture, which is more sensitive to fiscal funds than other industries and more in need of fiscal support and protection [25,26]. Theoretically, as the main capital input for sustainable agricultural economic development [27], fiscal agricultural expenditures have an important driving role. On one hand, it can directly promote sustainable agricultural economic development. On the other hand, it can indirectly drive sustainable agricultural economic development through agricultural industry integration. Its mechanism is shown in Fig 1.

#### 2.2.2 Direct influence path of fiscal agricultural expenditures on sustainable agricultural economic development

Theoretically, fiscal agricultural expenditures have a promoting effect on sustainable agricultural economic development, with four main paths of influence. First, improving material conditions of agricultural production. Through investing in the construction of seed breeding bases, farmland water conservancy, large-scale agricultural machinery facilities, agricultural product processing bases, warehousing, and other agricultural pre-production, production, and post-production service facilities, fiscal expenditures can increase agricultural productivity and enhance agricultural comprehensive productivity. Second, enhancing technological conditions for agricultural progress. By investing in the construction of agricultural research facilities such as agricultural experiments, agricultural education, and training, the technical level of agricultural production and labor force can be improved to break through the bottleneck of agricultural productivity [28]. Third, perfecting social conditions for agricultural development. Via supporting investment in rural roads, communication networks, power grids, and other agricultural social infrastructure can not only meet the needs of agricultural production and directly improve the efficiency of agricultural production, but it is also conducive to the rapid dissemination and transformation of agricultural scientific research results into actual productivity. Fourth, optimizing the financial conditions for agricultural investment. Supporting the development of rural inclusive finance by incentives, interest rate subsidies, tax concessions, and the establishment of credit guarantees, etc., fiscal expenditures can lead profit-tending financial resources to invest in agricultural production and satisfy the increasing demand for capital due to the agricultural industry upgrading. Therefore, this paper proposes the following hypothesis:

**Hypothesis 1.** The improvement in fiscal agricultural expenditures has a positive impact on sustainable agricultural economic development.

Further, the effect of fiscal agricultural expenditures on promoting sustainable agricultural economic development is changing dynamically. In the long run, China’s agriculture is in the process of a dynamic transition from traditional agriculture to modern agriculture. Along with the agricultural development stage upgrading, China’s supporting reforms of the fiscal institutional mechanism are constantly advancing, and the fiscal agricultural policies are constantly optimized in the process of transitioning from quantity to quality, from comprehensiveness to focus, and from a single path to multi-governance [29]. Meanwhile, the level of agricultural modernization is constantly improving, agricultural comprehensive development capacity is becoming stronger, and the investable fields and input paths of agriculture are constantly expanding, which in turn improves the rate of agricultural investment return. Therefore, the driving effect of fiscal agricultural expenditures on sustainable agricultural economic development will become more and more significant. In summary, this paper puts forward the following hypothesis:

**Hypothesis 2.** The impact of fiscal agricultural expenditures on sustainable agricultural economic development is a long-term dynamic process and shows a gradual upward trend with the continuous development of the agricultural economy.

#### 2.2.3 Indirect influence path of fiscal agricultural expenditures on sustainable agricultural economic development

In addition to its direct impact, fiscal agricultural expenditures may also indirectly promote sustainable agricultural economic development by deepening the integration of agricultural industry. Agricultural industry integration refers to the dynamic industrial innovation process of forming a new business model by taking agriculture as the basic foundation.

Agricultural industry integration becomes an innovative driving force for transforming the growth mode of the agricultural economy. It can break the clear boundaries between the original three industries, and then integrate and reorganize the advantageous factor resources of the three. Through agricultural industrial intersection and its related secondary and tertiary industries in terms of technology, products, services, and markets [30], agricultural industry integration can effectively embed capital, science and technology, information, talent, and other elements into the whole agricultural industry chain. Thus, it can promote the enhancement of the total factor productivity of agriculture [31], accelerate the transformation and upgrading of the agricultural industry, and improve the comprehensive strength of agriculture [32]. It deeply explores the diversified agricultural functions and expands the value-added space of agricultural industry in multiple dimensions [33], therefore more values and benefits that belong to the secondary and tertiary industries can be left in agriculture [34].

Usually, there are three paths for agricultural industry integration: the multi-directional extension of agricultural industry chain, the multi-dimensional expansion of industrial functions, and the integrated development of agricultural service industry. Fiscal agricultural expenditures can indirectly affect sustainable agricultural economic development through the above three paths.

First, vertical path: agricultural industry chain extension. This model continuously extends the forward and backward industry chain with agricultural production as the center and promotes the integrated operation of all links in the whole agricultural industry chain, such as agricultural production, processing, and circulation [35]. Among them, the processing industry leads the integrated development of agriculture, influencing agricultural economy and rural development by expanding the value-added space of agriculture [36,37]. Fiscal agricultural expenditures usually provide a convenient economic environment for such projects in diversified forms, for example, in the form of tax-based expenditures, implementing preferential policies on income tax for enterprises engaged in the primary processing of agricultural products; in the form of fiscal subsidies, expanding the pilot scope of the subsidies for the purchase of new agricultural machines, and granting preferences for the purchase of processing equipment for grain, cotton, oil, sugar and other important agricultural products.

Second, horizontal path: agricultural multifunctionality expansion. This model centers on a single agricultural economic function, expands agricultural multifunctionality such as culture, ecology, tourism, education, etc., and forms a new industrial form dominated by leisure agriculture and rural tourism industry, which helps to keep the tertiary value derived from agriculture still in agriculture and reduce regional inequality [38,39]. Fiscal agricultural expenditures often take a variety of ways to support the integration of agriculture and rural tourism, for example, to build first and then subsidize, award in lieu of subsidy, work in lieu of relief, and other ways to help rural tourism develop, and gradually improve the rural roads, car parks, water and electricity supply, network, and other supporting facilities for agricultural life.

Third, cooperative path: agricultural service development. The model aims to achieve the coordinated development of agriculture and agricultural service industry, mainly providing agricultural technology consulting, agricultural insurance, and other specialized support services for the whole agricultural chain. Socialized service delivery in all aspects of agricultural production can effectively improve the efficiency of agricultural production [40,41]. Fiscal agricultural expenditures generally promote the integration in the form of portfolio inputs, for instance, through government purchase, guiding farmers’ cooperatives, industry associations, leading enterprises, and other service organizations to undertake agricultural production services; through investment in establishing agricultural development funds, supporting for agricultural production socialized service projects.

Based on the above, this paper proposes the following research hypothesis:

**Hypothesis 3.** Agricultural industry integration plays an intermediary role between fiscal agricultural expenditures and sustainable agricultural economic development.

## 3. Empirical research design

### 3.1 Data source

The research sample of this paper contains panel data from 31 provinces in mainland China from 2008 to 2020. The data mainly come from the “China Statistical Yearbook”, the “China Agricultural Yearbook”, the “China Population and Employment Statistical Yearbook”, the “China Rural Financial Services Report”, the “China Financial Yearbook”, as well as the Provincial Statistical Yearbooks, and China Three Rural Databases. At the same time, the interpolation method is used to make up for the very few missing data. To ensure the reliability and comparability of the data, this paper deflates all monetary volume indicators using various price indices with 2008 as the base period and takes the logarithms of the scale variables.

### 3.2 Variable selection

#### 3.2.1 Explained variable

The explained variable is sustainable agricultural economic development (*SAED*). This paper adopts a broad connotation of agriculture, including agriculture, forestry, animal husbandry, and fishery, and thus selects rural per capita value added of agriculture, forestry, animal husbandry, and fishery to measure the level of sustainable agricultural economic development in each province. The value added of agriculture, forestry, animal husbandry, and fishery is the final result of the production activities of agriculture, forestry, animal husbandry, and fishery in a certain period, expressed in monetary terms, representing the value added from the goods produced and the supporting activities provided by their services, so this indicator has the most direct representation.

#### 3.2.2 Explanatory variable

The core explanatory variable is fiscal agricultural expenditures (*FAE*). In this paper, rural per capita expenditure on agriculture, forestry, and water affairs is used to indicate the level of China’s fiscal expenditure on agriculture. Expenditure on agriculture, forestry, and water affairs is a new caliber used to calculate the scale of fiscal support for agriculture since 2007, including expenditure on agriculture and rural areas, forestry and grassland, water conservancy, comprehensive rural reform, inclusive financial development and consolidation of poverty alleviation and rural revitalization, which is the most comprehensive reflection of the importance attached by the Chinese government to "Three Rural Issues", and plays a key role in stimulating, guiding, and supporting agricultural development.

#### 3.2.3 Control variables

To effectively observe the influence effect of fiscal agricultural expenditures on sustainable agricultural economy, it is necessary to control the influence factors as much as possible. This paper selects control variables from agricultural production, economic, policy, and environmental levels respectively.

First, agricultural production factors. Agricultural human capital level (*Edu*), and agricultural mechanization level (*Pow*) are selected as representative indicators, specifically with rural per capita schooling years and rural per capita agricultural machinery power to measure.

Second, economic factors. This paper takes local economic development level (*Gdp*) and rural financial development level (*Loan*) as representative indicators, which are expressed by per capita gross regional product and rural per capita agricultural loans, respectively.

Third, policy factors. Referring to Wang et al. [42], this paper chooses the regime environment score (*Regi*) as the measurable indicator. The higher the score, the higher the market-oriented allocation proportion of regional economic resources, the smaller the local government’s ability to control resources, and the greater the ability to create a more favorable business environment as well as a more fair and transparent market environment for agricultural market players.

Fourth, environmental factors. Agricultural disaster level (*Disa*) is chosen as a proper indicator.

The specific measurement method and basic statistical information of each variable are presented in the descriptive statistics table, as shown in Table 1.

**Table 1:** Variable definition and descriptive statistics.

Variable Type	Variable Name	Symbol	Variable Definition	Obs	Mean	Std	Min	Max
Explained Variable	Sustainable agricultural economic development	*SAED*	ln (Rural per capita value added of agriculture, forestry, animal husbandry, and fishery)	403	0.668	0.276	0.199	1.668
Explanatory Variable	Fiscal agricultural expenditures	*FAE*	ln (Rural per capita fiscal expenditures on agriculture, forestry, and water affairs)	403	0.165	0.141	0.031	0.855
Control Variable	Agricultural human capital level	*Edu*	Rural per capita schooling years	403	7.608	0.830	3.819	9.801
	Agricultural mechanization level	*Pow*	ln (Rural per capita gross power of agricultural machinery)	403	1.628	0.816	0.326	6.186
	Economic development level	*Gdp*	ln (Per capita gross regional product)	403	2.573	1.512	0.804	8.656
	Rural financial development level	*Loan*	ln (Rural per capita agricultural loans)	403	1.890	1.474	0.011	9.572
	Regime environment score	*Regi*	1-Regional fiscal expenditures/Gross regional product	403	0.728	0.202	-0.379	0.913
	Agricultural disaster level	*Disa*	Agricultural disaster area/Sown area	403	0.172	0.132	0.006	0.689

Note: Descriptive statistics values refer to their values before taking logarithms.

### 3.3 Model setting

#### 3.3.1 Benchmark regression model

To verify the relationship between fiscal agricultural expenditures and sustainable agricultural economic development, based on the analysis of the previous theoretical mechanism, this paper constructs a time and province two-way fixed effect model for research:

$$SAED_{it}=\alpha_{0}+\alpha_{1}FAE_{it}+\sum Control_{it}+\mu_{i}+\lambda_{t}+\varepsilon_{it}$$ (1)

*i* denotes province, *t* denotes year; *SAED*_*it*_ denotes sustainable agricultural economic development; *FAE*_*it*_ denotes fiscal agricultural expenditures; *∑Control*_*it*_ denotes a series of control variables; *μ*_*i*_ denotes province-fixed effects; *λ*_*t*_ denotes time-fixed effects; and *ε*_*it*_ denotes a disturbance term. In Eq (1), the coefficient of interest in this paper is *α*_*1*_ if the estimated value is greater than 0, it means that fiscal agricultural expenditures promote sustainable agricultural economic development.

#### 3.3.2 Panel quantile regression model

To further characterize the dynamic evolution of the marginal effect of fiscal agricultural expenditures in the process of sustainable agricultural economic development, following Powell [43], this paper uses a panel quantile model to estimate Eq (1):

$$Q_{SAED_{it}}=\gamma (\tau )FAE_{it}+\varphi (\tau )\sum Control_{it}$$ (2)

*τ* denotes the corresponding quantile. *Q*_*SAEDit*_ denotes the level of sustainable agricultural economic development under the corresponding quartile. *γ(τ)* denotes the regression coefficients of the core explanatory variable under the corresponding quantile, and *φ(τ)* denotes the coefficients of each control variable under the corresponding quantile. In addition, 10%, 30%, 50%, 70%, and 90% are selected as quartiles in this paper.

## 4. Empirical results

### 4.1 Baseline regression: Average impact

The article first carries out the Hausman test on the model, and the test results in P = 0.00002, indicating that it is significant at a 1% significance level, thus rejecting the original hypothesis of applying random effect model, that is, this model applies a fixed effect model. Based on this, the impact of fiscal agricultural expenditures on sustainable agricultural economic development with the full sample is shown in Table 2.

**Table 2:** Baseline regression results.

Variable	(1) *SAED*	(2) *SAED*	(3) *SAED*	(4) *SAED*	(5) *SAED*
*FAE*	0.6806***	0.4638***	0.1381**	0.1381*	0.1381***
	(14.5305)	(9.3380)	(2.3799)	(1.9698)	(2.9422)
*Edu*		0.1043*	0.0140	0.0140	0.0140
		(1.9564)	(0.4106)	(0.4856)	(0.5723)
*Pow*		0.3025***	0.2057***	0.2057***	0.2057***
		(4.8274)	(3.3628)	(3.5412)	(6.7274)
*Loan*		-0.0219	0.0301	0.0301	0.0301
		(-1.2880)	(1.1497)	(1.3059)	(1.2309)
*Gdp*		0.4068**	0.3025**	0.3025*	0.3025***
		(2.6940)	(2.2038)	(1.9817)	(3.8972)
*Regi*		0.2239	0.5614**	0.5614*	0.5614***
		(0.9086)	(2.1884)	(2.1508)	(2.8280)
*Disa*		-0.1579**	-0.0657	-0.0657	-0.0657
		(-2.1103)	(-1.1981)	(-1.2527)	(-1.3978)
Individual fixed effect	Yes	Yes	Yes	Yes	Yes
Time fixed effect	No	No	Yes	Yes	Yes
Observations	403	403	403	403	403
Adj.R^2^	0.7016	0.8066	0.9680	0.9680	0.9680
Cluster Robust Standard Error	province Clustering	province Clustering	province Clustering	province-year Clustering	Heteroskedasticity Robust Standard Error

Note

***, **, and * represent statistical significance at 1%, 5%, and 10%, respectively. Number in the parentheses is the Cluster-adjusted t-statistic. Adj.R^2^ is the adjusted R^2^.

Columns (1)–(3) of Table 2 report the impact of fiscal agricultural expenditures (*FAE*) on sustainable agricultural economic development (*SAED*) without control variables, with control variables, and with two-way fixed effects, respectively, all of which are clustered to the provincial level using Cluster Robust Standard Error, referring to Chen and Zhang [44]. From the estimation results, it can be seen that fiscal agricultural expenditures exert a significant positive effect on agricultural economic development. From column (1), the regression coefficient of *FAE* without control variables is 0.6806 and significant at a 1% level. From the results of columns (2) and (3), the core coefficient is still positive after adding control variables and two-way fixed effects, and the *R*^*2*^ value increases gradually, which indicates that the positive effect of *FAE* is significant and stable. From the final result of column (3) as the benchmark regression, it can be seen that the average impact effect of the core explanatory variable is 0.1381 and significant at the 5% level, for every 1% increase in fiscal agricultural expenditures inputs, the sustainable agricultural economy grows by 0.1381%, while other factors are kept constant. The above results indicate that fiscal agricultural expenditures play a significant role in incentivizing sustainable agricultural economic development. Accordingly, Hypothesis 1 proposed in this paper is verified.

Referring to the error term treatment of Sun et al. [45], based on the setting of column (3), the error term treatments of columns (4) and (5) are adjusted from province clustering to province-year clustering and adopting Heteroskedasticity Robust Standard Error, respectively. The regression results under the above settings show that the benchmark regression is not affected by the change in the treatment of the error terms, and the estimated coefficient of the core explanatory variable *FAE* remains significantly positive.

As for control variables, the sign of each variable’s coefficient is consistent with the expectation. Among them, the regression coefficient of *Pow* is significantly positive at the 1% level, indicating that the increase in agricultural mechanization level can effectively promote the growth of sustainable agricultural economy. The estimated coefficient of *Gdp* is positive and significant at the 5% level, suggesting that the better the regional economy develops, the more conducive to the development of the agricultural economy. The estimated coefficient of *Regi* is positive and significant at the 5% level, implying that provinces with a more favorable institutional environment can provide better development support for the agricultural economy, and the agricultural economy can grow by 0.56% for every 1-point increase in the institutional environment. In addition, the regression coefficients of the variables of *Edu*, *Loan*, and *Disa* are not significant, which means that factors such as agricultural human capital, rural finance development, and agricultural natural environment cannot influence the agricultural economy significantly in the research stage of this paper.

### 4.2 Panel quantile regression: Marginal impact

**Fig 2:**
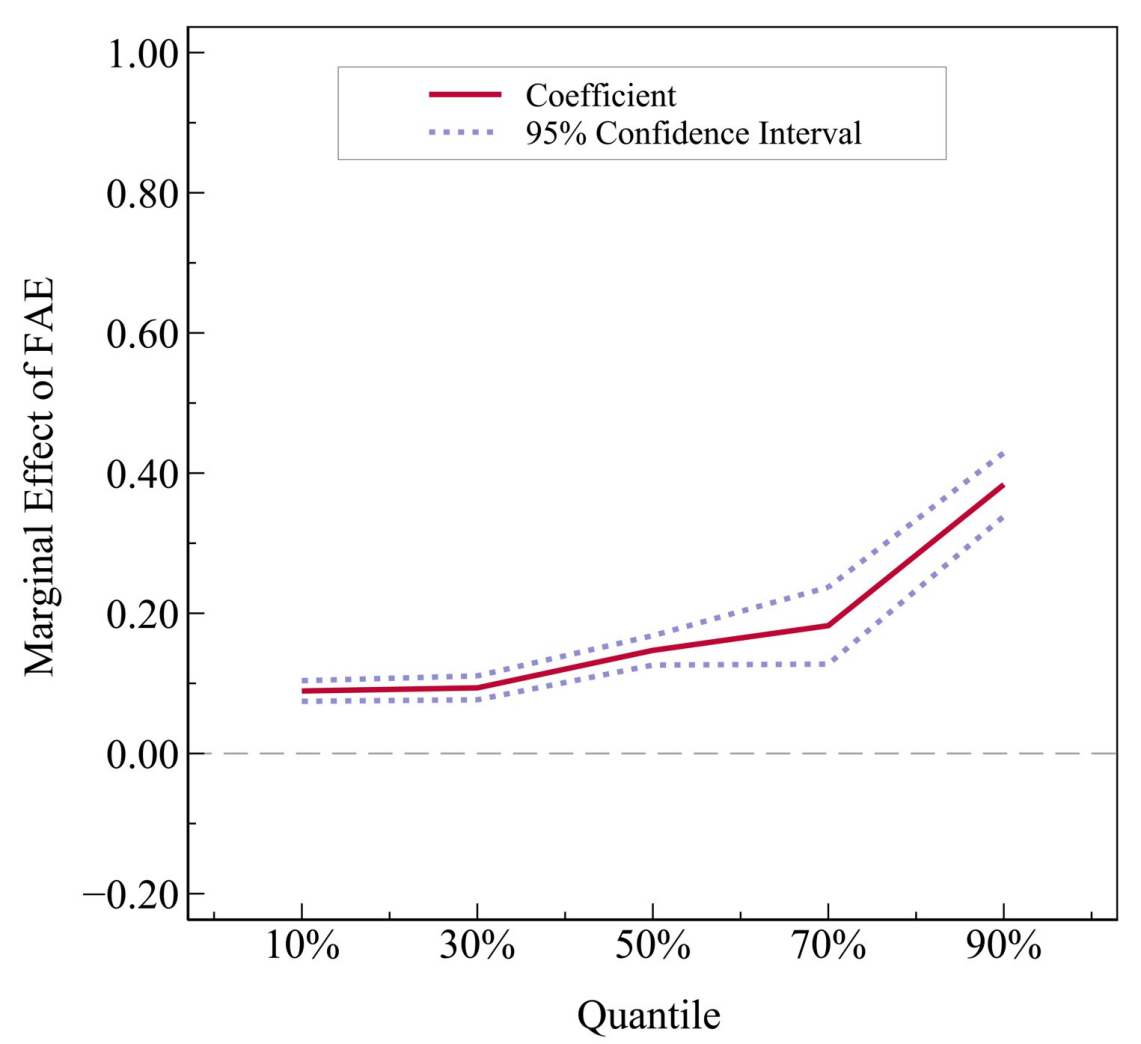
Marginal effect of fiscal agricultural expenditures.

Based on Table 2, to further analyze the dynamic evolution trajectory of fiscal agricultural expenditures’ marginal impact under different stages of sustainable agricultural economic development, this paper adopts the panel quantile regression technique for parameter estimation and gives the marginal effect of fiscal agricultural expenditures and its evolution trend when all control variables are considered, as shown in Fig 2 and Table 3.

**Table 3:** Panel quantile regression results.

Variable	(1) 10%	(2) 30%	(3) 50%	(4) 70%	(5) 90%
*FAE*	0.0891***	0.0936***	0.1471***	0.1824***	0.3837***
	(11.8734)	(10.7616)	(13.7272)	(6.5182)	(16.6138)
*Edu*	0.0068	-0.0925***	-0.0224*	0.1150***	0.1394***
	(0.2460)	(-9.8889)	(-1.7106)	(15.0258)	(3.0725)
*Pow*	0.1921***	0.2791***	0.3321***	0.4234***	0.1560***
	(40.6085)	(49.0299)	(14.3969)	(37.8465)	(3.6433)
*Loan*	0.0557***	0.1727***	0.1271***	0.0819***	0.0766***
	(12.1528)	(21.5729)	(11.5490)	(8.0850)	(7.3511)
*Gdp*	-0.1210***	0.0245***	0.0967***	-0.0266	0.0134
	(-10.6589)	(2.9993)	(5.6207)	(-0.9006)	(0.3177)
*Regi*	0.7714***	1.1348***	1.0540***	1.0194***	0.6264***
	(9.2648)	(55.2524)	(16.4258)	(11.5560)	(6.2961)
*Disa*	-1.3244***	-0.7290***	-0.2971***	-0.1556***	-0.2827***
	(-18.3562)	(-13.6065)	(-5.8040)	(-6.8009)	(-3.8232)
Individual fixed effect	Yes	Yes	Yes	Yes	Yes
Time fixed effect	Yes	Yes	Yes	Yes	Yes
Observations	403	403	403	403	403

Note

***, **, and * represent statistical significance at 1%, 5%, and 10%, respectively. Number in the parentheses is the t-statistic, which is obtained by simulating 1000 estimates of the corresponding variable with the Markov Chain Monte Carlo method (MCMC), and the following quantile regressions are the same as here.

Combined with Table 3 and Fig 2, it can be seen that in different stages of sustainable agricultural economic development (*SAED*), the marginal impact of fiscal agricultural expenditures (*FAE*) is consistently positive and shows a rising dynamic evolutionary trend. It indicates that in the stage of a higher level of sustainable agricultural economic development, the promotion effect of fiscal agricultural expenditures on the agricultural economy becomes more obvious. Accordingly, Hypothesis 2 is verified.

As for control variables, it can be observed from Table 3 that with sustainable agricultural economic development, the marginal effect of *Edu* ranges from insignificant to negatively significant to positively significant, which may be attributed to the following reason: In the region with a relatively weak base of the agricultural economy, a large number of rural laborers, especially young and middle-aged laborers with a high level of literacy, have flowed to the non-agricultural industry, and the higher the level of agricultural human capital in the region, the more serious the loss of human capital, the lower the stock of agricultural human capital, the more constraints on the improvement of agricultural production capacity, thus producing a negative impact [46]; with the gradual increase in the level of sustainable agricultural economic development, the agricultural sector’s ability to absorb labor and high-level human capital has gradually increased, and the degree of rural human capital loss has been weakened, and its promotional effect on the agricultural economy has begun to gradually appear. Similarly, *Gdp* is not entirely consistent with the expected sign and significance at all stages of agricultural economic development, which may be related to the various economic development stages of each region’s industrial planning layout and industrial structure adjustment and other factors, resulting in the non-consistency of regional agricultural economic growth and overall economic growth. Except for the above two variables, the marginal effects of other control variables throughout the stage of agricultural economic development are in line with expectations.

The above results indicate that sustainable agricultural economy is realized by the parallel and alternating drive of multidimensional factors in the process of long-term development, and the drive of any single factor cannot replace the play of the common effectiveness of multiple factors.

### 4.3 Robustness test

The results of benchmark regression and quantile regression show that China’s fiscal agricultural expenditures effectively promote sustainable agricultural economic development, and its marginal effect shows a dynamic evolutionary trend of continuous increase with the development of sustainable agricultural economy. To eliminate the interference of confounding factors on the estimation results, this paper mainly starts by replacing the explained variable, lagging the explanatory variables by one period, and changing the estimation method to confirm the above conclusions. The results are shown in Table 4.

**Table 4:** Robustness test results.

Method	Variable	(1) Full Sample	(2) 10%	(3) 30%	(4) 50%	(5) 70%	(6) 90%
Replacing explained variable	*FAE*	0.1498**	0.0261	0.0361***	0.0564***	0.0634***	0.3586***
		(2.4914)	(0.6793)	(6.2618)	(5.7128)	(41.7099)	(60.3300)
	Adj.R^2^	0.9639					
Lagging explanatory variables by one period	*L1_FAE*	0.1176*	0.0151***	0.0892***	0.0978***	0.1105***	0.2050***
		(1.9446)	(2.8850)	(11.9991)	(17.0394)	(33.6189)	(106.2971)
	Adj.R^2^	0.9978					
Changing estimation method	*FAE*	0.1381***	0.0443	0.1780*	0.1804*	0.1898*	0.4481***
		(2.9574)	(0.2374)	(1.8684)	(1.7012)	(1.8507)	(3.2512)
	Adj.R^2^	0.9680					
Control		Yes	Yes	Yes	Yes	Yes	Yes
Individual fixed effect		Yes	Yes	Yes	Yes	Yes	Yes
Time fixed effect		Yes	Yes	Yes	Yes	Yes	Yes
Observations		403	403	403	403	403	403

Note

***, **, and * represent statistical significance at 1%, 5%, and 10%, respectively. Number in the parentheses is the t-statistic. Adj.R^2^ is the adjusted R^2^.

#### 4.3.1 Replacing explained variable

This paper adopts the per capita value added of primary industry as a substitute to measure the level of sustainable agricultural economic development, and the values are also treated in logarithmic terms. The value added of primary industry refers to the final results of all resident units in a certain area engaged in production activities of primary industry in a certain period calculated according to market prices, including the value added of agriculture, forestry, animal husbandry, and fishery, excluding the value added of agriculture, forestry, animal husbandry, and fishery services. The regression results confirm that the basic conclusions still hold.

#### 4.3.2 Lagging explanatory variable by one period

In this paper, all explanatory variables including control variables are treated with one lag. The reason is that there is a time cost in the turnover of fiscal agricultural expenditures, the output cycle of inputting into agricultural production is long, and there is a time lag effect in fiscal agricultural expenditures. The regression results are consistent with the original regressions.

#### 4.3.3 Changing estimation method

To avoid the problem of sample selectivity bias and further improve the consistency and validity of parameter estimation, this paper refers to Ma and Huang [47] to estimate the parameters by Bootstrap self-sampling method. The regression results show that the benchmark regression conclusions are robust.

### 4.4 Heterogeneity test

#### 4.4.1 Examination of subgroups based on benchmark regression

To explore the average impact of fiscal agricultural expenditures on sustainable agricultural economic development among different samples, this paper respectively groups the samples based on different dimensional criteria for regression and uses Fisher’s Permutation Test to carry out the Between-group coefficient difference test, the results are shown in Table 5.

**Table 5:** Heterogeneity test results.

Variable	Geographic Location			Industrial Structure	
	(1) Eastern	(2) Central	(3) Western	(4) High	(5) Low
*FAE*	0.1111**	0.1986	0.1094	0.3104***	0.1051
	(2.3053)	(1.7540)	(0.7410)	(3.1217)	(1.3542)
Control	Yes	Yes	Yes	Yes	Yes
Individual fixed effect	Yes	Yes	Yes	Yes	Yes
Time fixed effect	Yes	Yes	Yes	Yes	Yes
Observations	143	104	156	182	221
Adj.R^2^	0.9695	0.9691	0.9667	0.9740	0.9825
Between-group coefficient difference	Eastern–Central -0.0875 (0.1500)	Central–Western 0.0892 (0.2000)	Eastern–Western 0.0017 (0.4800)	High–Low 0.2050*** (0.0000)	

Note

***, **, and * represent statistical significance at 1%, 5%, and 10%, respectively. Number in the parentheses for Between-group coefficient difference is the p-value, others are the t-statistics. Adj.R^2^ is the adjusted R^2^.

First, regional heterogeneity. Referring to the National Bureau of Statistics Statistical System and Classification Standards, this paper divides the sample into three groups of Eastern, Central, and Western regions for regression according to geographic location. From the empirical results in columns (1)–(3) of Table 5, it can be seen that *FAE* in the eastern region significantly promotes agricultural economic growth at the 5% level, while the empirical results in the central and western regions are not significant, but the results of the between-group test show that the differences in the effect of fiscal agricultural expenditures among the three sample groups are not significant.

Second, industrial structure heterogeneity. The industrial structure is expressed by the ratio of the value added of primary industry to GDP, and this paper takes the average value of this index as the basis for division and divides the whole sample into two groups with a high share of primary industry and a low share of primary industry for comparative analysis of heterogeneity. The results from columns (4) and (5) of Table 5 show that the high share group is significant at the 1% level, while the regression coefficient of the low share group is not significant. The significant difference in the coefficients between the groups indicates that there is a difference in the effect of fiscal agricultural expenditures under this subgroup. The possible reason for this is that the high proportion of primary industry means that the agricultural economy is more important in economic development, and thus the sample group has superior conditions that are more favorable for the effectiveness of fiscal funds in the dimensions of agricultural policy support and agricultural production factors.

#### 4.4.2 Examination of subgroups based on panel quantile regression

**Fig 3:**
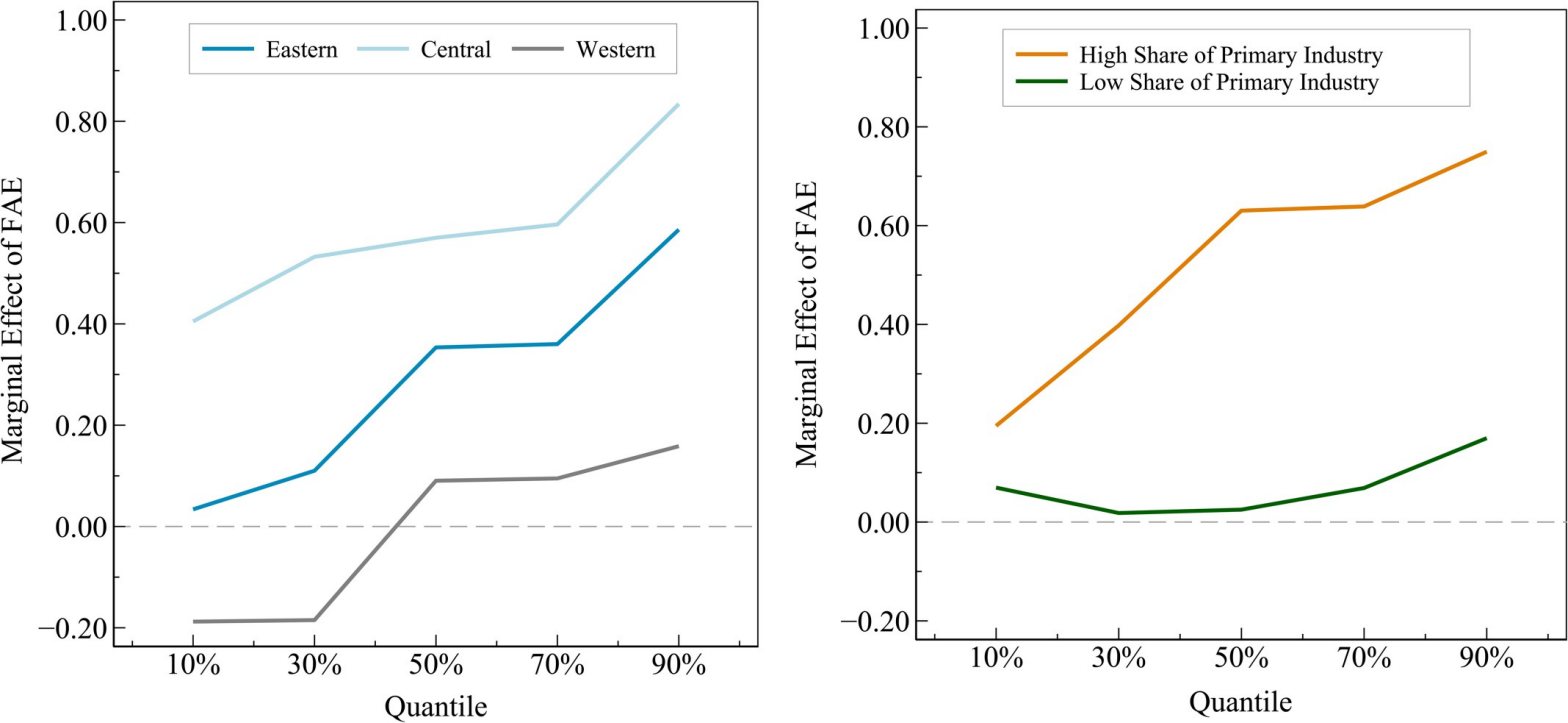
Heterogeneity analysis of panel quantile regression.

To more intuitively and accurately observe the dynamic evolution trajectory of the marginal effect of fiscal agricultural expenditures at different stages of sustainable agricultural economic development under different subgroups, this paper continues to adopt the same subgroups and standards based on the heterogeneity analysis of the benchmark model to further examine the heterogeneity characteristics of the panel quantile regression model, and the test results are shown in Fig 3.

As can be seen in Fig 3, firstly, under different stages of sustainable agricultural economic development, the marginal effect of fiscal agricultural expenditures shows a dynamic evolution trend of gradually rising in each subgroup, which further verifies the setting of Hypothesis 2. Secondly, under each subgroup, the marginal effects of fiscal agricultural expenditures in each sub-sample form their different evolutionary trajectories, which confirms that heterogeneity exists objectively. In addition, from the regression results of geographic location grouping, the heterogeneity test results of the benchmark regression are not significant, while the heterogeneity characteristics of the panel quantile regression are very obvious, which indicates that compared with the static average effect analysis of the benchmark regression model, the dynamic marginal effect analysis of the panel quantile regression model is more helpful in presenting the full picture and the overall characteristics of the sample data.

Specifically, in the industrial structure grouping, the test results and related reasons are consistent with the heterogeneity analysis of the above benchmark regression. While in the geographic location grouping, the results show significant differences. Compared with the western region, the impact of fiscal agricultural expenditures on sustainable agricultural economic development in the central region is greater, probably because the central region has more suitable natural endowments such as water, light, soil, heat, and so on for food crop production than the western region. Meanwhile, compared with the eastern region, the central region is more dependent on the agricultural economy for its economic development and its agricultural policy environment, it is more conducive to sustainable agricultural economic development. Thus, the central region can develop sustainable agricultural economy and release the potential effectiveness of fiscal agricultural expenditures to a greater extent.

## 5. Further mechanism test

The above empirical study confirms the positive promotion effect of fiscal agricultural expenditures on sustainable agricultural economic development from both static average impact and dynamic marginal impact. Then, does the integration of agricultural industry play a significant role as an important path of fiscal agricultural expenditures to promote sustainable agricultural economic growth? This section uses the mediating effect model to test the mechanism of fiscal agricultural expenditures affecting sustainable agricultural economic development.

### 5.1 Mediating effect model

Based on the theory that fiscal agricultural expenditures will promote sustainable agricultural economic growth through agricultural industry integration, this paper establishes the following mediating effect model based on Eq (1) according to Baron and Kenny [48], and Wen [49]:

$$AII_{it}=\beta_{0}+\beta_{1}FAE_{it}+\sum Control_{it}+\mu_{i}+\lambda_{t}+\varepsilon_{it}$$ (3)

$$SAED_{it}=\theta_{0}+\theta_{1}FAE_{it}+\theta_{2}AII_{it}+\sum Control_{it}+\mu_{i}+\lambda_{t}+\varepsilon_{it}$$ (4)

*AII*_*it*_ denotes the mediating variable, which is agricultural industry integration in this paper. The existence of the mediating effect of agricultural industry integration requires *α*_*1*_ significant in Eq (1), where the mediating mechanism test mainly focuses on *β*_*1*_ and *θ*_*2*_, if both are significant, further testing of *θ*_*1*_ is required, if *θ*_*1*_ is the same significant, then it indicates a partial mediating effect, otherwise, it indicates a full mediating effect. If *β*_*1*_ and *θ*_*2*_ at least one of the two is not significant, then it is necessary to carry out the Sobel test, and if the results are significant, then it indicates that there is a mediating effect, otherwise, it indicates that there is no.

### 5.2 Measurement and descriptive statistics of mediating variable

The mediating variable is agricultural industry integration (*AII*). Considering the research of Li and Lu [50], and Hao and Liu [51], this paper combines the connotation of agricultural industry integration, constructs a comprehensive evaluation index system of it, and utilizes the entropy value method for measurement. The evaluation system selects three primary and six secondary indicators, as shown in Table 6.

**Table 6:** Comprehensive evaluation indicator system for agricultural industry integration.

First-Level Index	Second-Level Index	Direction
Agricultural industry chain extension	Agro-industry income/Rural population	Positive
	Gross power of agricultural processing machinery /Gross power of agricultural machinery	Positive
Agricultural multifunctionality expansion	Leisure agriculture business income/Rural population	Positive
	Facility agriculture area/Cultivated land area	Positive
Agricultural service development	Gross value of agricultural, forestry, livestock, and fishery services /Rural population	Positive
	Number of specialized farmers’ cooperatives /Rural population	Positive

The descriptive statistics of the mediating variable are shown in Table 7.

**Table 7:** Variable definition and descriptive statistics.

Variable Type	Variable Name	Symbol	Variable Definition	Obs	Mean	Std	Min	Max
Mediating Variable	Agricultural industry integration	*AII*	Agricultural Industry Integration Index	403	0.190	0.095	0.018	0.492

### 5.3 Trend analysis of agricultural industry integration

#### 5.3.1 Time distribution characteristic

**Fig 4:**
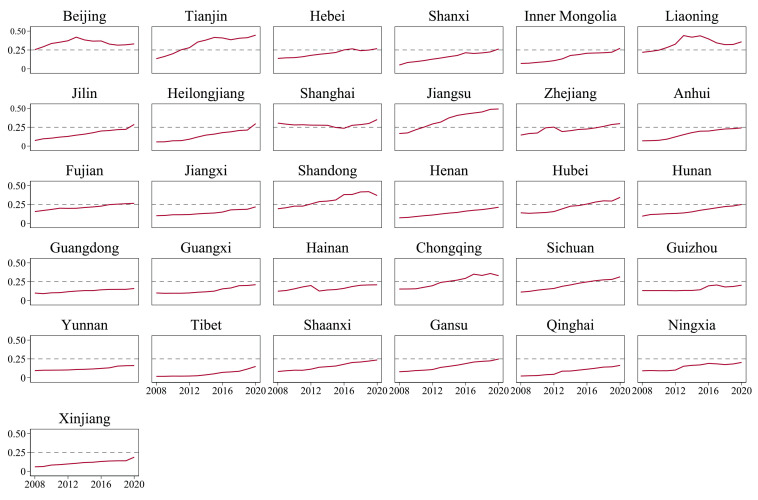
Level of agricultural industry integration in 31 provinces of China during 2008–2020.

This part presents the specific measurement results of the agricultural industry integration level in 31 provinces of China from 2008 to 2020, and the temporal distribution of each province can be seen in Fig 4.

Overall, the results of agricultural industry integration level measurement show several obvious features. Firstly, from the sample value domain, there is a significant imbalance in agricultural industry integration level among different provinces, with most of the provinces having an agricultural industry integration index lower than 0.25, and only nine provinces, such as Beijing, Tianjin, Liaoning, etc., have a higher level than 0.25 in certain years. Secondly, from the perspective of the overall development trend, the agricultural industry integration level in most of China’s provinces shows a continuous upward trend, with only a few regions having fluctuating tendencies, such as Beijing, Liaoning, Shandong, and so on. Thirdly, in terms of the overall development trend, most provinces in China show a continuous upward trend in agricultural industry integration level, with only a few regions such as Beijing, Liaoning, and Shandong experiencing fluctuating trends.

#### 5.3.2 Spatial distribution characteristic

**Fig 5:**
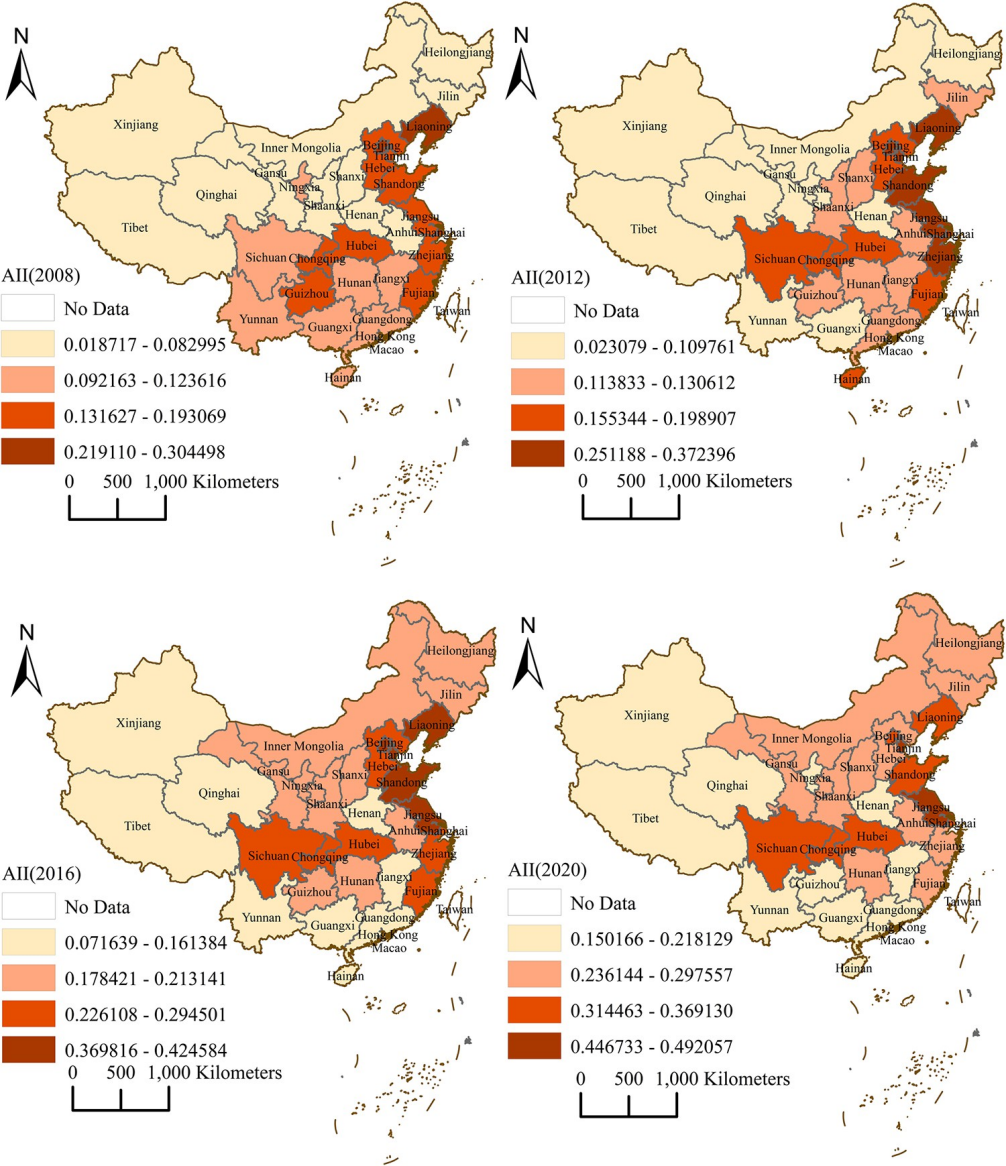
Spatial distribution of agricultural industry integration level in China. Note: The base map came from United States Geological Survey (https://apps.nationalmap.gov/services/), the map boundary of this study has not been changed and it does not represent the true borders of administrative regions of China. Cartographic software: ArcGIS.

This section examines the spatial distribution characteristics of agricultural industry integration levels in 31 provinces of China in 2008, 2012, 2016, and 2020, ranking the Agricultural Industry Integration Index of all provinces for each year, as shown in Fig 5.

In the spatial distribution of this paper, the different color of the different provinces in the same year stands for the relative level of agricultural industry integration nationwide, and the different color of the same province in the different year indicates the relative level change of the province in the nationwide. It can be observed that provinces with relatively high levels of agricultural industry integration are mainly concentrated in the eastern coastal region, and the relative level of agricultural industry integration in the north-eastern provinces has risen faster than that in the south-eastern provinces, while the relative integration level in the western region is consistently weaker than that in the eastern and central regions. The results suggest that the eastern regions are generally more favorable to agricultural industry integration because of their superior level of economic development, fiscal input, and technology. In addition, as central and western regions, the industry integration levels of Sichuan, Chongqing, and Hubei have been higher than those of other provinces, indicating that the region attaches higher importance to agricultural industry integration, starts agricultural industry integration at an earlier starting point, and has a good foundation for agricultural industry integration.

#### 5.3.3 Regional distribution characteristic

**Fig 6:**
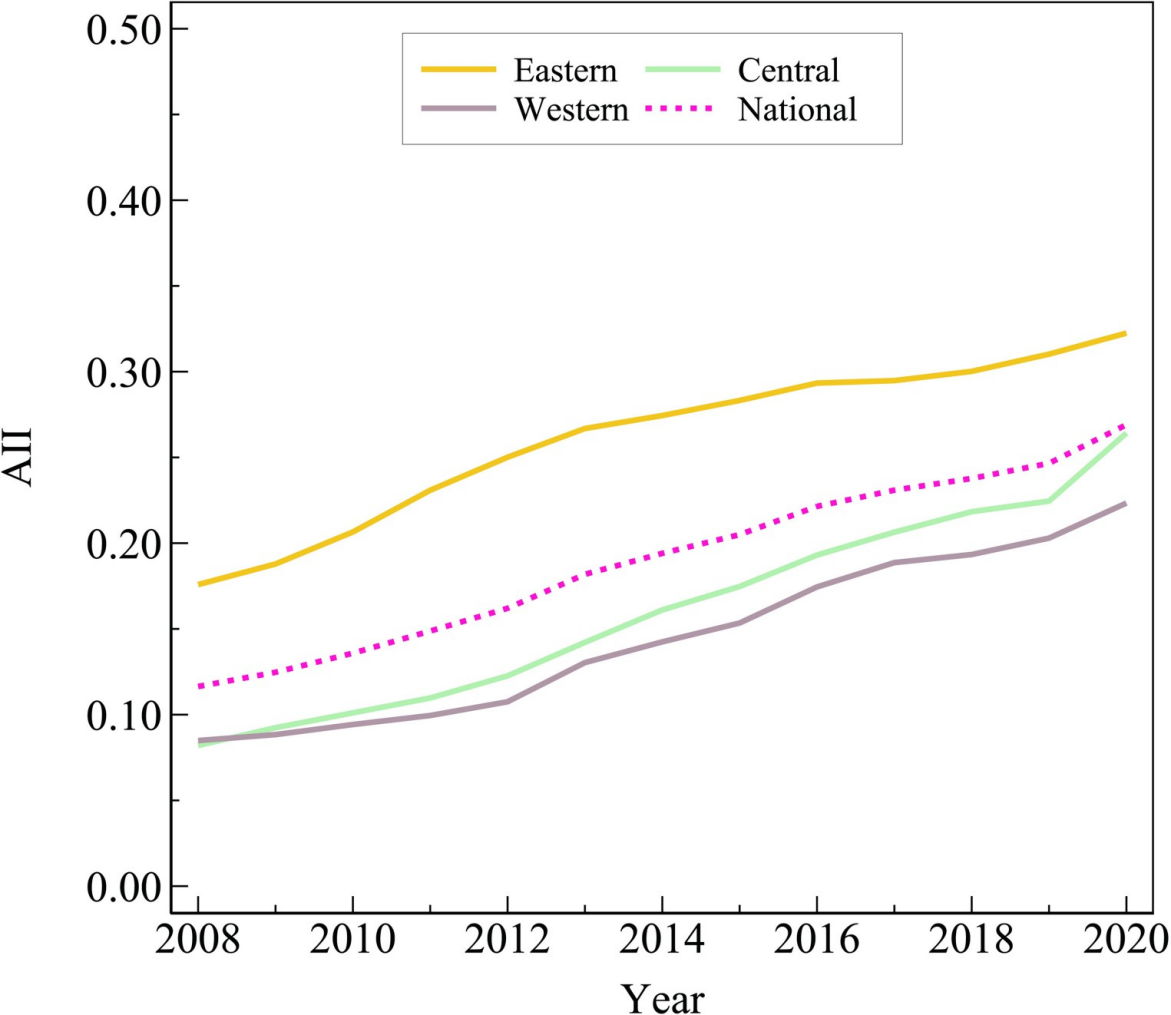
Regional characteristics of agricultural industry integration.

This section summarizes the agricultural industry integration level in a regional classification, as shown in Fig 6.

From the perspective of regional distribution characteristics, from 2008 to 2020, all regions’ agricultural industry integration index shows the evolution trend of "continuously rising", and the eastern level is much higher than the national average level, while the central level and the western level are lower than the national average level. This phenomenon may come from the fact that the eastern region has developed economic level, and people’s demand for agricultural industry integration is higher; meantime it possesses richer capital, science and technology, human resources, and other resources, thus the resource base of agricultural industry integration is much higher ***than*** that of other regions. Besides, the integration level of the central region in 2008 was almost the same as that of the western region, and the trend of the integration index growth rate has been faster than that of the western region in recent years, which indicates that the central region may have adjusted the policy orientation and increased the investment in agricultural industry integration.

### 5.4 Mediating effect results

To verify whether agricultural industry integration plays an intermediary role in the process of fiscal agricultural expenditures driving sustainable agricultural economic development, this section conducts a transmission mechanism test, the results are shown in Table 8.

**Table 8:** Mediating effect results.

Panel A			Panel B: Sobel Test	
Variable	(1) *AII*	(2) *SAED*	Effect	(3) *SAED*
*FAE*	0.2594*	0.1250***	Indirect Effect	0.0131*
	(1.9448)	(3.1203)		(1.6601)
*AII*		0.0506***	Direct Effect	0.1250***
		(3.1865)		(3.1203)
Control	Yes	Yes	Control	Yes
Individual fixed effect	Yes	Yes	Individual fixed effect	Yes
Time fixed effect	Yes	Yes	Time fixed effect	Yes
Observations	403	403	Observations	403
Adj.R^2^	0.7744	0.9689	Mediate Proportion	9.51%

Note

***, **, and * represent statistical significance at 1%, 5%, and 10%, respectively. Number in the parentheses is the z-statistic. Adj.R^2^ is the adjusted R^2^.

From columns (1) and (2) of Table 8, it can be seen that the impact coefficient of fiscal agricultural expenditures (*FAE*) on agricultural industry integration (*AII*) is significant, and the impact coefficient of fiscal agricultural expenditures (*FAE*) and agricultural industry integration (*AII*) on sustainable agricultural economic development (*SAED*) is also significant, which indicates that the mediating effect exists; however, after the introduction of mediating variable, the impact of fiscal agricultural expenditures (*FAE*) on sustainable agricultural economic development (*SAED*) is still significant. Furthermore, in the Sobel test, the result of column (3) shows that the mediating effect accounts for 9.51%.

In summary, fiscal agricultural expenditures can promote sustainable agricultural economic development through agricultural industry integration, thus Hypothesis 3 is established.

## 6. Conclusions and implications

### 6.1 Conclusions

With the world facing an unprecedented global food security crisis, exploring the way to sustainable agricultural economic development is thus likely to be of profound significance. This paper has leveraged real-world evidence from China to estimate whether and how fiscal agricultural expenditures affect sustainable agricultural economic development. The results show that there is a positive dynamic impact of fiscal agricultural expenditures on sustainable agricultural economy, and agricultural industry integration plays an intermediary role between them, which provides a new theoretical perspective and empirical evidence for improving fiscal agricultural policies.

This paper contributes to the literature in three ways. First, we provide strong evidence to prove that fiscal agricultural expenditures promote sustainable agricultural economic development, which is consistent with the hypothesis and indirectly responds to the debate surrounding the practical effect of fiscal agricultural expenditures in previous literatures [21–24]. We also find significant regional heterogeneity in the impact of fiscal agricultural expenditures, and the impact is stronger in central areas and areas with a high proportion of primary industry. These results affirm the effectiveness, necessity, and heterogeneity of sustained investment in fiscal agricultural expenditures.

Second, our study adds the dynamic marginal effect test of fiscal agricultural expenditures unlike previous literatures mainly focusing on the static average effect [13–15]. The results show a dynamic evolution trend of gradual increase with the upgrading of the agricultural development stage, providing empirical support for the future long-run fiscal support policy of gradually expanding expenditures on agriculture.

Finally, the research fills the gap of influencing mechanism of fiscal agricultural expenditures based on the existing literatures, providing preliminary evidence that agricultural industry integration plays a mediating role in fiscal agricultural expenditures promoting sustainable agricultural economy through three paths of agricultural industry chain extension, multi-functional agricultural expansion, and agricultural service industry development. These results may guide the future adjusting direction of fiscal expenditures on agriculture.

One concern about the research is that there is a certain degree of data constraint on the mediating variable during the mechanism test, which is reflected in the fact that the comprehensive index system of agricultural industry integration cannot be represented as multidimensional as it is due to the data availability of some sub-indicators and the research preference. Future work could be devoted to obtaining a more scientific mediating variable by constructing a more systematic index system of agricultural industry integration with an extension of the data collection method.

### 6.2 Policy implications

The above findings suggest three potential directions for policy improvement to further promote sustainable agricultural economy.

Firstly, a long-term fiscal investment mechanism is needed, considering its continued contribution to agriculture. To be specific, the scale of fiscal agricultural expenditures should be gradually expanded to ensure the sustainability of investment. Meanwhile, agriculture-related financial funds need to accelerate the integration and management process and improve the efficiency of use. In addition, it is necessary to strengthen the fiscal leverage to guide more private capital into agricultural projects.

Secondly, fiscal agricultural expenditure policies should be differentiated in view of their heterogeneity. Specifically, fiscal investments in agriculture should increase with the stage upgrading to meet the higher input demand at the higher stage. Besides, fiscal expenditures should be invested in projects with prominent regional and industrial characteristics and more significant production increases in the western area due to its poor agricultural production capacity and investment efficiency. Moreover, the central government should strengthen its support for provinces with a high proportion of primary industry.

Finally, the intermediary role of agricultural industry integration ought to fully be released. Concretely, local finance at all levels is required to prioritize the fund’s arrangement to support agricultural industry integration, especially for the central and western regions with poor levels of agricultural industry integration. Additionally, the forms of fiscal support for agriculture are supposed to be diversified like tax incentives and financial awards, and lead to more private capital in agricultural industry integration projects. What’s more, considering the multi-dimensional connotation of sustainable agricultural economy, fiscal support for agricultural industry integration should involve many aspects of agricultural production, society, and environment. In particular, investment in sustainable development of agricultural environmental dimension should be strengthened, which is the basis of other dimensions of sustainable agricultural economy.
